# The Role of SGLT2 Inhibitors in Vascular Aging

**DOI:** 10.14336/AD.2020.1229

**Published:** 2021-08-01

**Authors:** Le Liu, Yu-Qing Ni, Jun-Kun Zhan, You-Shuo Liu

**Affiliations:** ^1^Department of Geriatrics, The Second Xiangya Hospital, Central South University, Changsha, Hunan 410011, China.; ^2^Institute of Aging and Age-related Disease Research, Central South University, Changsha, Hunan 410011, China.

**Keywords:** vascular aging, sodium-dependent glucose transporters 2 inhibitor, arterial stiffness, endothelial cells, vascular smooth muscle cells

## Abstract

Vascular aging is defined as organic and functional changes in blood vessels, in which decline in autophagy levels, DNA damage, MicroRNA (miRNA), oxidative stress, sirtuin, and apoptosis signal-regulated kinase 1 (ASK1) are integral thereto. With regard to vascular morphology, the increase in arterial stiffness, atherosclerosis, vascular calcification and high amyloid beta levels are closely related to vascular aging. Further closely related thereto, at the cellular level, is the aging of vascular endothelial cells (ECs) and vascular smooth muscle cells (VSMCs). Vascular aging seriously affects the health, economy and life of patients, but can be delayed by SGLT2 inhibitors through the improvement of vascular function. In the present article, a review is conducted of recent domestic and international progress in research on SGLT2 inhibitors,vascular aging and diseases related thereto, thereby providing theoretical support and guidance for further revealing the relationship between SGLT2 inhibitors and diseases related to vascular aging.

## 1.Introduction

The increasing number of people with diabetes mellitus (DM) has rendered vascular diseases, one of the numerous complications thereof, more apparent. DM can cause major vascular diseases (e.g., cardiovascular and cerebrovascular diseases), microvascular diseases (e.g., retinopathy, kidney diseases, and neuropathy), and vascular diseases of the foot (such as diabetic foot). Vascular aging is defined as organic and functional changes in blood vessels, and with the aging of the human body, blood vessels gradually lose their original functions, which results in arterial stiffness and a faster pulse wave transmission rate [1-3]. As a specific type of organic aging, vascular aging accounts for age-related changes in the vascular system [4]. Although the physiological aging caused by increasing age cannot be intervened, premature aging caused by stress factors, such as DM and hypertension, is likely to be prevented and treated through interventions. Diabetic patients have prominent vascular aging problems owing to severe vascular complications, which can easily lead to serious vascular diseases in the heart, brain and other parts of the body. Accordingly, diabetic patients can experience serious health problems, impairing physical health and adding to the financial burden of the families thereof. Hence, improving vascular function and delaying vascular aging in DM patients is of considerable import. The limited understanding of the occurrence and progressof DM-induced vascular aging has led to unsatisfactory results being achieved with regard to the prevention thereof in DM patients. Recently, the emergence of SGLT2 inhibitors has aroused the interest of an increasing number of scholars, who have applied said inhibitors for the purpose of improving vascular function and delaying vascular aging. As a new type of hypoglycemic agent, SGLT2 inhibitors have been widely studied for the protective effects thereof on blood vessels, with several studies also exploring the role of said inhibitors in the prevention of vascular aging. These studies are reviewed in this paper, before a summary is provided on the recent advances in the role of SGLT2 inhibitors in improving vascular function and delaying vascular aging caused by DM.

SGLT2 inhibitors [5] are a series of novel oral hypoglycemic drugs with anti-diabetic properties, including Dapagliflozin, Canagliflozin, Tofogliflozin, Empagliflozin, *etc*. (Table 1), which are capable of lowering blood sugar by inhibiting glucose reabsorption in the proximal tubules of the kidney and excreting glucose into urine. SGLT2I- is unique in the capabilities thereof to lower blood sugar and inhibit insulin secretion independent of insulin, with no increase to the risk of hypoglycemia. For these reasons, SGLT2 inhibitors are a promising treatment of type 2 DM (T2DM). In addition to lowering blood sugar, SGLT2 inhibitors can also inhibit the expression of SGLT2 in the renal proximal tubules to promote sodium diuresis and glucose permeable diuresis [6, 7], increase urate excretion to reduce uric acid [8], lower blood pressure [9] by excreting sodium and reducing weight and uric acid, and improve the prognosis of heart failure [10]. Although newly developed, SGLT2 inhibitors have been broadly applied in clinic practice because of the aforementioned positive effects. Both domestic and international scholars have gradually shifted focus towards the anti-vascular aging effect of SGLT2 inhibitors.

In the present article, a review is conducted on the recent domestic and international progress in the research on SGLT2 inhibitors, vascular aging and diseases related thereto, thereby providing theoretical support and guidance for further revealing SGLT2 inhibitors and diseases relating to vascular aging.

**Table 1:** Three common SGLT2 inhibitors

	Canagliflozin	Dapagliflozin	Empagliflozin
Specification	100mg;300mg	5mg;10mg	10mg;25mg
Dosage	100mg daily, 300mg max	5mg daily, 10mg max	10mg daily, 25mg max
Half-life	100mg: 10.6 h, 300mg: 13.1 h	12.9h	12.4h
Time to reach peak plasma concentration	1h-2h	2h	1.5h
Oral bioavailability	65%	78%	70-90%
Protein binding	99%	91%	86.2%
Metabolism	UGT1A9, UGT2B4, CYP3A4	UGT1A9, CYP	UGT2B7, UGT1A3, UGT1A8, UGT1A9
Excretion	51.7% feces, 33% urine	75% urine, 21% feces	54.4% urine, 41.2% feces
Molecular formula	C24H25FO5S1/2H2O	C21H25CIO6·C3H8O2·H2O	C23H27ClO7
Volume of distribution	83.5L	118L	73.8L

## 2.The mechanism of vascular aging

**Figure 1.**
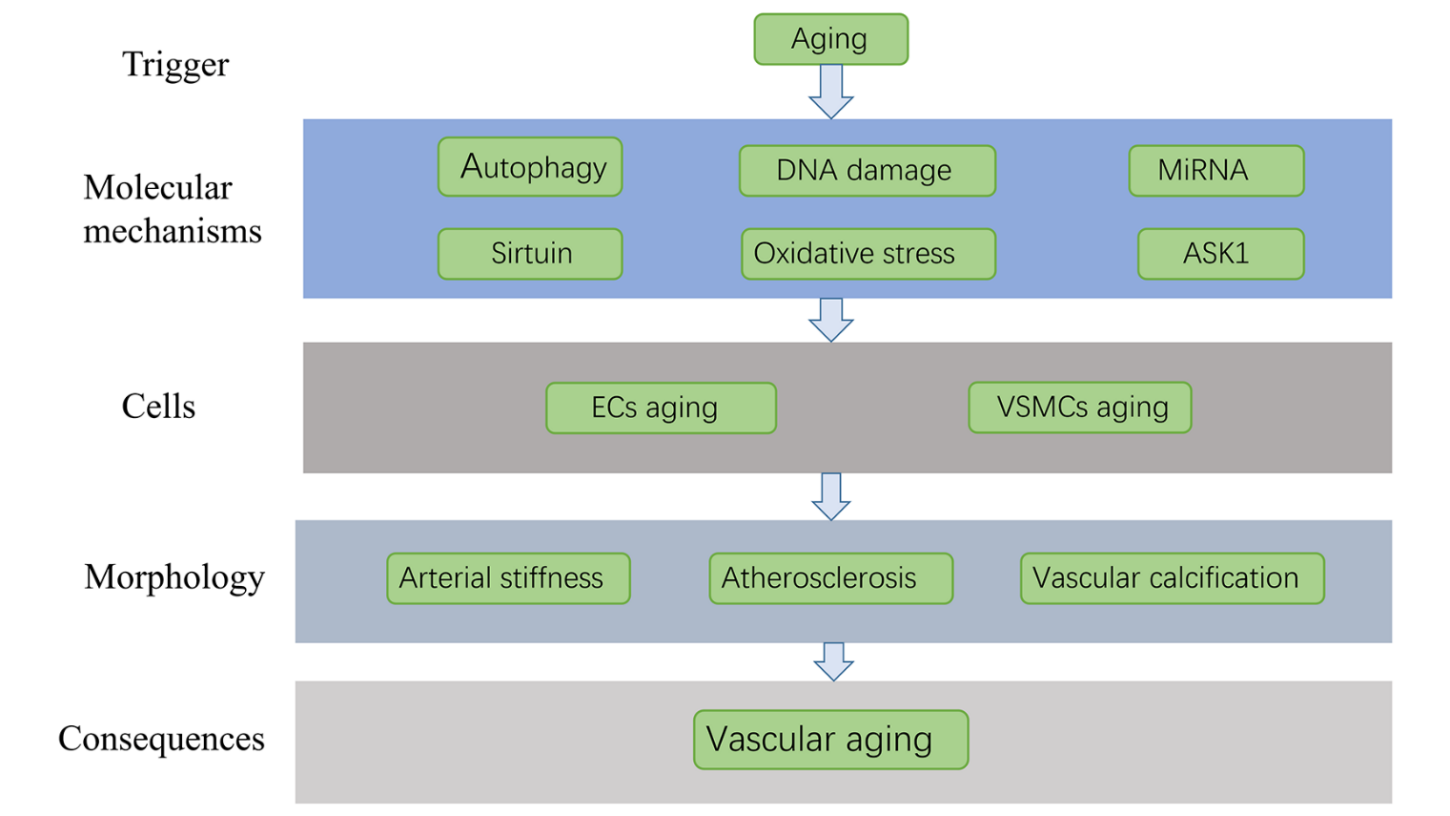
The mechanism of vascular aging.

The mechanism of vascular aging is considerably complicated. At the molecular mechanism level, decline in autophagy levels, DNA damage, miRNA, oxidative stress, sirtuin, and apoptosis signal-regulated kinase 1 (ASK1) are integral to vascular aging. Meanwhile, at the cellular level, the aging of vascular endothelial cells (ECs) and vascular smooth muscle cells (VSMCs) are closely related to vascular aging. Further closely related thereto, in terms of vascular morphology, are the increase in arterial stiffness, atherosclerosis, vascular calcification and high amyloid beta levels. In this part, the mechanism of vascular aging is discussed from the aforementioned three aspects (Fig. 1).

### 2.1 The molecular mechanism of vascular aging

Vascular aging usually starts from changes at the molecular level and is the result of the accumulation of multiple changes, being accelerated by the interaction of various factors such as inflammation, DNA damage and the decline of autophagy level.

### 2.1.1 Oxidative stress and inflammation

Closely linked to vascular aging, excessive oxidative stress and chronic inflammation can lead to endothelial dysfunction therein. Oxidative stress damages endothelial progenitor cells (EPC) and promotes cell aging [11]. Further, oxidative stress in the vasculature, characterised by an increase of ROS in the arteries, also contributes to decreased endothelium-dependent dilation (EDD) function, reduced NO bioavailability, and increased stiffness of large arteries. Chronic and low-grade inflammation has also been demonstrated to cause age-related ailments corresponding to endothelial function and exacerbate atherosclerosis. Circulatory markers of inflammation, CRP and IL-6 in particular, are positively correlated with aortic sclerosis and negatively correlated with elderly EDD [12].

### 2.1.2 MiRNAs

MiRNAs are also implicated in vascular aging. MiR-34a not only down-regulates Silent Information Regulator 2 homolog 1, but also promotes the expression of age-related pro-inflammatory secretory factors, thereby promoting VSMC aging [13]. Reports have also indicated that miR-23 cluster can regulate blood vessel formation [14], while miR-30e can inhibit proliferation and migration of VSMCs, promote the apoptosis thereof, and exert anti-atherosclerosis effects [15]. Moreover, by inhibiting autophagy of vascular endothelial cells and promoting cell apoptosis, miR-199a-5p has been revealed to damage vascular endothelial cells and aggravate vascular aging [16].

### 2.1.3 DNA damage

Further related to vascular aging, DNA damage includes many aspects, such as single- and double-stranded breaks, partial DNA deletion, and extrusions of DNA from the nucleus. Changes in the expression of DNA damage response proteins play an important role in this process [17]. The study found that DNA damage in the nuclei and mitochondria of VSMCs, ECs and other cells in older people was significantly worse than that in younger people [18-20]. As DNA damage becomes more and more serious and the repairing cascades are damaged, cell senescence and apoptosis occur [21].

### 2.1.4 Decline in autophagy levels

Autophagy is a way to regulate cell metabolism, and a decrease in the levels thereof are often accompanied by vascular aging. In the elderly, the expression of autophagy markers in arterial endothelial cells is reduced by 50% [22], while the expression thereof in the aorta of aged mice also demonstrated a reduction [23]. Autophagy is vital for VSMC function and survival. Defective autophagy in VSMCs can lead to upregulation of matrix metalloproteinase9, transforming growth factor β and chemokine (C-X-C) motif ligand 12 and promote postinjury neointima formation and atherogenesis,thereby inducing vascular aging [24]. By enhancing autophagy, the expression of autophagy markers can be restored, NO-mediated EDD can be saved by reducing oxidative stress, and the expression of inflammatory cytokines can be normalised[22]. Hence, autophagy is negatively related to vascular aging.

### 2.1.5 Sirtuin

Sirtuin 1 (SIRT1) is highly expressed in ECs, and a vital regulator of vascular remodeling in response to vascular endothelial growth factor (VEGF)-stimulated angiogenesis [25]. A close link exists between vascular aging and the decrease of SIRT1, in which the reduction of SIRT1 has been demonstrated to increase the senescence of vascular cells, up-regulate the expression of p21, and enhance vascular inflammation [26]. A significant reduction of the endothelial expression of SIRT1, eNOS activity and endothelium-dependent vasodilation of SIRT1 in aging mice has been exhibited, while the overexpression in endothelial cells could also enhance eNOS activity and endothelium-dependent vasodilation, revealing that SIRT1 could counteract vascular aging [27, 28]. Enhancing SIRT1 activity can also alleviate Ang II-induced VSMCs aging [29]. Sirtuin 3 (SIRT 3) is also related to vascular aging, with PDGF-induced migration of VSMCsbeing inhibited by activating mitochondrial deacetylase SIRT 3 [30].

### 2.1.6 ASK 1

With the ability to control intracellular apoptotic events, ASK1 is activated when cells are stressed and then mediates a wide range of intracellular responses [31]. ASK1 activation can result in endothelial cell apoptosis or autophagy, which can then result in atherosclerotic endothelial dysfunction and vascular aging [32, 33].

### 2.2 The mechanism of vascular aging at the cellular level

As the main cells of blood vessels, ECs and VSMCs may experience replicative aging and induced aging in the body. Replicative aging occurs after cell division due to the damage and repair of blood vessels, while induced aging is related to the accumulation of harmful components in the blood. The combination of aging of said cells eventually leads to vascular aging.

### 2.2.1 ECs

The endothelium is the innermost lining layer of vascular, whichseparates the blood from other surrounding tissues. Recent research has highlighted the importance of this semi-permeable membrane and the critical role thereof in maintaining normal vascular function. It is said that semi-permeable membrane enables substances such as nutrients and leukocytes to pass through the barrier, while also being able to secrete multiple mediators necessary for maintaining normal vascular function. This includes those thatreduce oxidative stress, improve vascular homeostasis, and promote normal cell growth. Endothelial dysfunction can significantly destroy the structure and function of blood vessels and is a critical cause and mechanism of vascular aging. In addition, pathophysiological changes in ECs senescence can be attributed to NO bioavailability changes, endothelium-derived hyperpolarising factor (EDHF) and Ca2+ signaling, increased endothelium permeability, impairment of vascular self-regulation, and reduction of ECs mitochondrial biogenesis. Meanwhile, increased ROS, cell cycle change, oxidative stress, altered Ca2+ signaling, hyperuricemia, and vascular inflammation participate in the pathophysiological process [34]. The senescence of endothelial cells can form a chain reaction, which reduces the production of NO by endothelial cells, produces more endothelin 1, aggravates oxidative stress, and promotes apoptosis [35]. EC senescence damages the normal function of blood vessels, promotes thrombosis, oxidative stress and atherosclerosis, and weakens the ability of blood vessels to self-regulate, all of which contribute to vascular aging [34].

### 2.2.2 VSMCs

VSMCs possess the function of maintaining vascular tone, normal blood pressure and normal blood flow distribution. These cells continuously absorb biochemical components from the blood, convert them into mechanical forces to stimulate the blood flow, and participate in all physiological and pathological changes in the blood vessel wall. VSMCs aging can increase arterial stiffness, promote the occurrence and development of oxidative stress and inflammation, aggravate vascular aging and increase the incidence of vascular diseases [36]. Notably, VSMC phenotypic conversion can promote vascular aging by increasing arterial stiffness, arterial pulse pressure and dilatation of conduit arteries [37]. Through the interleukin-1α-driven senescence-associated secretory phenotype and the priming of adjacent cells to a proatherosclerotic state, senescent VSMCs can also aggravate inflammation and oxidative stress, thereby promoting the occurrence and development of atherosclerosis[38].

### 2.3 The mechanism of morphological changes in vascular aging

As a result of vascular regulation by both genes and environment, vascular aging is often accompanied by changes in vascular structure and function, including increased arterial stiffness, atherosclerosis, and vascular calcification.

### 2.3.1 Arterial stiffness

Arterial stiffness is an important manifestation of vascular aging, with almost all the occurrences of vascular aging being accompanied by arterial stiffness in clinical practice. As arteries are more elastic in young individuals, the pulse wave velocity (PWV) thereof is slower, and the diastolic blood pressure is higher. With aging blood vessels, arterial stiffness increases, pulse wave velocity (PWV) increases, systolic blood pressure increases, and pulse pressure difference increases, which in turn results in various other diseases. Arterial stiffness has a close association with the progression of DM complications (namely nephropathy, retinopathy and neuropathy), involving multiple organs and systems. Large-artery stiffness will increase the left ventricular afterload [39, 40] and effectuate left ventricular hypertrophy [41] and coronary perfusion damage. Further, arterial stiffness has been demonstrated to be significantly correlated with the development of diabetic retinopathy (DR) [42], neuropathy in T2DM patients [43], production of proteinuria in diabetic patients, a decreased glomerular filtration rate (GFR) [44] and degenerative cognitive status [45].

### 2.3.2 Atherosclerosis

Atherosclerosis and vascular aging are inextricably linked and mutually affected. A variety of pathological processes are involved in the vascular dysfunction caused by atherosclerosis. This includes oxidative stress, inflammation and autophagy, which are all also mechanisms of vascular aging [46]. Moreover, the occurrence of atherosclerosis hardens and thickens the arterial wall, leads to stenosis of the blood vessel cavity, intensifies arterial stiffness and inflammation, and ultimately accelerates the aging of blood vessels.

### 2.3.3 Vascular calcification

Vascular calcification (VC) is a vitalsign of vascular stiffness and aging, which can increase PWV, systolic pressure, pulse pressure and left ventricular load [47]. In addition, the vascular calcification process seems to be related to lipid oxidation, senescence of endothelial cells and chronic inflammation, which all promote vascular aging [48]. Thus, a distinct correlation exists between VC and vascular aging.

### 2.3.4 High amyloid beta levels

Vascular dysfunction, as one of the most common pathological changes in Alzheimer's disease and a mechanism of vascular aging, can be caused when amyloid beta (Aβ) deposits in cerebral blood vessels [49]. The concentration of Aβ in the brain cells of rats increased with age in animal research [50], while in clinical studies, the circulating levels of Aβ40 in the elderly were higher than those in the young even in the absence of diabetes, stroke and other diseases [51]. Additionally, elevated circulating levels of amyloid β40 can also be found in patients with coronary heart disease [52].

## 3.The effect of SGLT2 inhibitor on vascular aging

**Figure 2.**
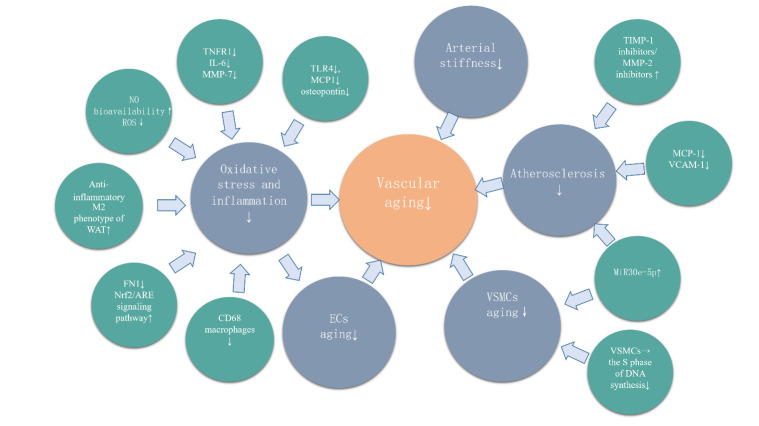
The effect of SGLT2 inhibitor on vascular aging.

As a new type of hypoglycemic agent, SGLT2 inhibitors have been established in clinical guidelines for a myriad of diseases. The anti- vascular- aging effect of SGLT2 inhibitor is evident (Fig. 2) and is discussed in the present article with respect to three aspects.

### 3.1 In vitro cell evidence of the effect of SGLT2 inhibitors on vascular aging

Empagliflozin and Dapagliflozin can mitigate endothelial inflammation induced by tumor necrosis factor alpha (TNFα) in vitro [53], in addition to increasing NO bioavailability and suppressing the generation of reactive oxygen species (ROS) induced by TNFα. Elevated ROS levels often suggest endothelial dysfunction [54], while NO is an endothelium-dependent relaxation factor, and the reduced bioavailability thereof contributes significantly to endothelial dysfunction. Hence, the SGLT2 inhibitors of Empagliflozin and Dapagliflozin directly attenuate endothelial aging by reducing ROS content and increasing NO bioavailability, thereby improving vascular function and delaying vascular aging. Further, the proliferation and migration of VSMCs that contribute to the development of arterial lesions and are closely related to vascular aging can be inhibited by SGLT2 inhibitors. As established by prior studies, Canagliflozin can reduce the proliferation and prevent the migration of rat aortic VSMCs in a concentration-dependent manner. Meanwhile, extremely high concentrations of Empagliflozin and Dapagliflozin have been demonstrated to moderately constrain the growth of VSMCs. When rat aortic VSMCs were treated with Canagliflozin for 24 hours, the level of heme oxygenase-1 (HO-1) protein exhibited a concentration-dependent increase, which produced CO and/or bilirubin to impede the proliferation and migration of VSMCs. Additionally, Canagliflozin can arrest the growth of VSMCs at the G0/G1 phase of the cell cycle and prevent VSMCs from entering the S phase of DNA synthesis, thereby majorly reducing the synthesis of VSMC DNA and restraining VSMC proliferation [55]. A study on the effect of Igligliflozin on the ECs in vitro of diabetic mice has further revealed that Ipragliflozin prevented endothelial dysfunction, and the mechanism was related to oxidative stress [56]. Ipragliflozin inhibited the production of ROS, in addition to reducing the expression of inflammatory molecules in the abdominal aorta of diabetic mice, such as monocyte chemotactic protein-1 (MCP-1), vascular cell adhesion molecule-1 (VCAM-1) and intercellular adhesion molecule-1 (ICAM-1) [56]. As elucidated by another prior study on proximal tubular human kidney cells, Canagliflozin treatment can reduce the levels of TNF receptor 1 (TNFR1), IL-6, matrix metalloproteinase 7 (MMP-7) and fibronectin 1 (FN1), exhibiting the inflammation reversion effect of Canagliflozin [57].

### 3.2 Animal evidence of the effect of SGLT2 inhibitors on vascular aging

A study on 10-week-old female diabetic mice highlighted that, through mechanisms not related to blood pressure reduction, Empagliflozin could reduce systemic arterial stiffness [58]. Similarly, Lee DM and Battson ML et al. observed that endothelial dysfunction in diabetic mice treated with Dapagliflozin for 2 months could be improved [59]. SGLT2 inhibitors have also been demonstrated to obviously delaythe aging of VSMCs in diabetic animal models, restore the function of damaged VSMCs to the level of the control group, and return the smooth muscle function to normal values through high-dose stimulation [59, 60]. Moreover, research has shown that SGLT2 inhibitors could prevent the development of endothelial dysfunction and delay vascular aging in animal models of T2DM by reducing glucose toxicity, oxidative stress and inflammation, and improving the viability of hyperglycemic endothelial cells [61]. Empagliflozin treatment could also substantially ameliorate oxidative stress in the heart of T2DM mice. Here, Empagliflozin lowered the activity of NADPH oxidase to inhibit the generation of the oxidation products thereof, namely, NOX4 (the major NADPH oxidase isoform in cardiomyocytes), in the myocardial tissue of diabetic rats. Further, Empagliflozin inhibited oxidative stress in the myocardium, which could potentially be attributed to the Nrf2 translocation being promoted to the nucleus and the Nrf2/ARE signaling pathway being activated [62]. In diabetic mice, Dapagliflozin could prevent oxidative stress through downregulating the expression of receptor for advanced glycation end products (RAGE)-induced NADPH oxidase in lens epithelial cells (LECs) via the inactivation of glucose transporters (GLUTs) and a reduction in ROS generation [63]. Another study demonstrated that Empagliflozin could prevent obesity-related chronic inflammation in high-fat-diet-induced obese (DIO) mice by inhibiting the accumulation of M1 polarised macrophages, inducing the expression of anti-inflammatory M2 phenotype of white adipose tissue (WAT) and macrophages in the liver, and reducing plasma TNFα levels [64]. The anti-inflammation effect of SGLT2 inhibitors could also be achieved by blocking the accumulation of CD68 macrophages and the expression of toll like receptor 4 (TLR4), monocyte chemotactic protein 1 and osteopontin [65]. Notably, SGLT2 inhibitors also have a significant effect on atherosclerosis. A study assessed the effects of long-term use of Empagliflozin on the indicators and parameters associated with atherosclerosis and the development thereof in apolipoprotein E gene knockout [Apo-E (-/-)] mice aortas. The results illustrated that the total cholesterol, fasting blood glucose, heart rate and blood pressure (DBP) of the mice treated with Empagliflozin were lower than those of the control group. Histomorphometry revealed that the decrease of the progression rate of atherosclerotic lesions in the Empagliflozin-treated mice group was nearly statistically significant, and the lumen area expanded by approximately 50%. For these reasons, by reducing hyperlipidemia and hyperglycemi, Empagliflozin improved the main hemodynamic parameters and slowed the progress of atherosclerosis [66]. Nasiri-Ansari and Dimitriades et al. also explored the effects of long-term treatment with Canagliflozin on the indicators and parameters related to atherosclerosis and the development thereof in the aortas of [Apo-E (-/-)] mice. An observation was made that Canagliflozin slowed the progress of atherosclerosis by down-regulating the expression of MCP-1 and VCAM-1, thereby reducing hyperlipidemia, hyperglycemia, and inflammation. Moreover, Canagliflozin enhanced the stability of atherosclerotic plaques by increasing the ratio of inhibitors of metalloproteinase-1 (TIMP-1)/matrix metalloproteinase-2 (MMP-2) at the mRNA level [67]. Short-term Luseogliflozin treatment could also attenuate the progression of atherosclerosis and normalise the expression of inflammation-related genes, but exerted no effect on the expression of lipid metabolism-related genes [68].

### 3.3 Clinical evidence of the effect of SGLT2 inhibitors on vascular aging

In a study of 32 patients with T2DM, findings were made that Dapagliflozin could considerably reduce carotid femoral artery pulsation (VPc-f) in T2DM subjects, suggesting that Dapagliflozin might alleviate long-term arterial stiffness [69]. Regardless of changes in blood pressure, Dapagliflozin treatment could drastically reduce carotid-femoral pulse wave velocity (cfPWV). A further study applied Empagliflozin, metformin, a combination of Empagliflozin and metformin, and a placebo to 10 T1DM patients separately, with the results illustrating that Empagliflozin was more effective than metformin in improving arterial stiffness. Additionally, the combined use of Empagliflozin and metformin exhibited a better performance than the single use of the two drugs. Empagliflozin can reduce arterial stiffness by not acting on nitric oxide (NO), inflammation or oxidative stress, which are factors well-known for the effect thereof of improving endothelial function. Thus, an assumption can be made that the mechanism underlying the arterial stiffness prevention effect of Empagliflozin is unique (independent of endothelial function), and may lie in the direct activation of specific receptor signaling pathways [70]. In a study of 16 patients with T2DM, intervention with Dapagliflozin notably increased arterial endothelium-dependent vasodilation and improved systemic endothelial function [71]. Further research has also shown that Canagliflozin of a concentration of 10 μm in the plasma of diabetic patients exhibits the effect of inhibiting VSMCs proliferation [72]. SGLT2 inhibitor Dapagliflozin can also improve vascular function and delay vascular aging in diabetic patients by acting on miRNA, specifically, by enhancing the expression of miR30e-5p and reducing the expression of miR199a-3p [73].

## 4. The role of SGLT2 inhibitors in vascular aging-related diseases

### 4.1 CVDs

Vascular aging in the cardiovascular system is prevalent in diabetic patients. CVDs, such as hypertension and coronary heart disease, are often closely related to DM, and pose a significant challenge to the treatment of T2DM. Antidiabetic drugs that reduce the risk of major cardiovascular events are undoubtedly a major advancement, with the newly developed SGLT2 inhibitors being able to reduce a variety of recognised cardiovascular risk factors, exert positive effects on the cardiovascular system, and lower the incidence of cardiovascular events. To be specific, SGLT2 inhibitors can promote glucose (calorie) excretion into urine thereby having the effect of controlling calories and weight, despite a slight compensatory increase in appetite and food intake [74, 75]. The natriuretic and diuretic effects of SGLT2 inhibitors can also help reduce arterial blood pressure [76, 77] and serum uric acid levels [78]. Moreover, during treatment with SGLT2 inhibitors, the increase in the hematocrit and blood concentration of patients could primarily be ascribed to the enhancement of red blood cell production. After diabetic patients started treatment with the SGLT2 inhibitor Dapagliflozin, the erythropoietin levels thereof increased and reached a plateau within 2 to 4 weeks. Simultaneously, the reticulocyte count increased, followed by hemoglobin and hematocrit [79]. SGLT2 inhibitors can also increase serum magnesium, potassium and phosphate concentrations, but the effect of elevated electrolytes on cardiovascular disease remains unclear. [80, 81]. 7020 T2DM patients with CVD were randomly assigned into several groups in the EMPA-REG OUTCOME trial and were treated with 10 mg or 25 mg Empagliflozin or a placebo once a day. The median duration of treatment was 2.6 years. The results illustrated that, compared with the placebo, Empagliflozin substantially reduced the risk of CVD-induced death, the mortality from any cause, and the hospitalisation rate due to heart failure. At the same time, Empagliflozin was correlated with slight reduction in weight, waist circumference, uric acid levels, and systolic and diastolic blood pressure. However, more patients in the Empagliflozin group had genital infections and uremia, and the patients treated with Empagliflozin suffered a higher risk of stroke. Notably, these points require verification through further research [82]. The CANVAS project combined data from two projects (CANVAS and CANVAS-R [Canagliflozin Cardiovascular Assessment Study-Renal]), including 10142 patients with T2DM and high-risk CVDs. Participants were randomly assigned to receive Canagliflozin or a placebo and followed up for an average of 3.6 years (median 2.4 years). The study found that the combined endpoint of cardiovascular death, non-fatal myocardial infarction, or non-fatal stroke in patients treated with Canagliflozin was 14% lower than that of the control group, which was a significant difference [83]. A multinational study compared the heart failure and death rates between patients newly treated with any SGLT2 inhibitor and patients taking other blood glucose-lowering drugs in 6 countries/regions. The results demonstrated that treatment with SGLT2 inhibitors could substantially reduce the incidence of heart failure and patient mortality. The evaluation of a wider range of populations with cardiovascular risks in this study suggested that the vast majority (87%) had no established CVDs, and the incidence of heart failure and mortality in these people also decreased. The results indicated that low-risk patients could benefit from SGLT2 inhibitors in the same way as high-risk patients [84].

### 4.2 DR

The vascular aging of the retina in diabetic patients is usually more serious than that in people without diabetes. DR is a diabetic microvascular complication [85] commonly detected in T1DM and T2DM patients, which can seriously diminish the vision and quality of life of patients, and even lead to blindness in severe cases [86]. Early diabetic vascular diseases are often accompanied by vascular hyperperfusion. As revealed in a study conducted by Grunwald et al., the blood flow in the retina of poorly controlled patients with T1DM and retinopathy was 23 % higher than that of normal people [87]. Hence, improving retinal vascular aging in diabetic patients is of considerable significance, and is an effect possessed by SGLT2 inhibitors. A clinical study involving 59 diabetic patients reported that, compared with patients treated with a placebo, the retinal capillary flow (RCF) of the patients treated with Dapagliflozin was significantly reduced after 6 weeks, and the retinopathy thereof was improved. Moreover, Dapagliflozin treatment has been demonstrated to prevent changes in the structure of retinal arterioles [88]. Notably, due to scarcity of research, the effect of SGLT2 inhibitors on DR requires further exploration.

### 4.3 Diabetic nephropathy

Diabetic nephropathy is a common microvascular complication in patients with T1DM and T2DM, and also one of the main causes of end-stage renal disease worldwide, leading to heavy health care costs related to dialysis and transplantation. This can significantly diminish the quality of life of patients and burden the families thereof [89]. Therefore, delaying renal vascular aging in diabetic patients is of significant import. SGLT2 inhibitors have been shown to have a positive therapeutic effect on diabetic nephropathy. A study revealed that treatment with Empagliflozin for 4 weeks could obviouslyreduce hyperglycemia, HbA1c, and the expression levels of oxidative stress markers AGEs, RAGE, 8-hydroxydeoxyguanosine (8-OHdG), and macrophages in patients with diabetic nephropathy. In this case, the presence of 8-OHdG and L-fatty acid-binding protein often indicated renal tubular damage. In this study, a suggestion was that although Empagliflozin did not directly reduce proteinuria, kidney damage was reduced by lowering the level of urinary 8-OHdG and L-fatty acid-binding protein. Additionally, the expression of monocyte chemoattractant protein-1, intercellular adhesion molecule-1, transforming growth factor-lysosome and connective tissue growth factor, which is usually up-regulated in diabetic kidneys, was inhibited by Empagliflozin [90]. A further study observed that the SGLT2 inhibitor Dapagliflozin improved hyperglycemia in T1DM Akita mice and delayed the development of diabetic nephropathy. To a large extent, Dapagliflozin inhibited the occurrence and development of renal interstitial fibrosis, inflammation, oxidative stress and apoptosis, thereby preventing the kidney pathology of the mice from worsening. By inhibiting the oxidative stress induced by hyperglycemia, SGLT2 inhibitors can improve diabetic nephropathy [91]. In a study of male DB/DB mice, 10 weeks of Empagliflozin treatment slowed the progression of diabetic nephropathy by attenuating the up-regulation of pro-fibrotic gene markers, fibulin, and transforming growth fact-beta induced by DM, and reducing the levels of several molecular and histological indicators of fibrosis. Yet, Empagliflozin was exhibited to be unable to suppress the increase of urine markers of renal tubular damage, renal hypertrophy or glomerulosclerosis [92]. Meanwhile, a clinical study established that Canagliflozin 100 or 300 mg/d could delay the progression of kidney diseases in patients with T2DM for more than 2 years compared with glimepiride [93]. A further study observed that, after a median follow-up time of 2.62 years, the risks of renal failure and cardiovascular events in patients with T2DM and kidney disease treated by the SGLT2 inhibitor Canagliflozin were lower than those in patients treated by a placebo [94]. The combined use of SGLT2 inhibitors and GLP-1 receptor agonists could also reduce proteinuria in patients with diabetic nephropathy, shorten the time to double serum creatinine, and delay the occurrence of end-stage renal disease [95]. In addition, a study on the combined use of SGLT2 inhibitor Dapagliflozin and irbesartan found that the combination could significantly reduce proteinuria, improve renal function parameters and the levels of RAGE, inflammation and oxidation markers, and attenuate renal histopathological changes. As a a promising option for the treatment of diabetic nephropathy, the combination exhibited a more significant protective effect on renal function and structure than independent use [96].

### 4.4 Cerebrovascular diseases

The human brain is considerably sensitive to the amount of perfusion, and common risk factors such as aging, smoking and hyperlipidemia will lead to cerebrovascular aging and serious cerebrovascular diseases. A complicated relationship exists between SGLT2 inhibitors and cerebrovascular aging-related diseases. Through meta-analysis, SGLT2 inhibitors have been shown to increase the incidence of stroke [97]. This result was unexpected, as SGLT2 inhibitors could improve the well-known risk factors for cerebrovascular diseases, including hypertension [98, 99], high weight [100], low HDL cholesterol levels [101] and high triglyceride levels [102]. In addition, several other studies on SGLT2 inhibitors and stroke have demonstrated that SGLT2 inhibitors did not increase the risk of stroke [103, 104]. Hence, further in-depth studies are required to explore the relationship between the two, with the risk of stroke in patients using SGLT2 inhibitors possibly being related to stroke subtypes [105].

## 5. Conclusion

In summary, as a new blood sugar lowering drug, SGLT2 inhibitors can effectively and safely lower blood sugar levels, redress the deficiencies of other blood sugar lowering drugs to a certain extent, and meet the needs of more T2DM patients. SGLT2 inhibitors also have promising prospects in improving vascular function and delaying vascular aging. Additionally, SGLT2 inhibitors can delay the aging of endothelial cells and smooth muscle cells, reduce inflammation and oxidative stress, regulate the expression of MicroRNA and prevent the occurrence and development of atherosclerosis, and can improve blood vessel stiffness and aging. At present, the potential effects and complex mechanisms of SGLT2 inhibitors have not been fully explored, and further basic and clinical research is required.
